# Stability and in vitro digestibility of beta‐carotene in nanoemulsions fabricated with different carrier oils

**DOI:** 10.1002/fsn3.862

**Published:** 2018-10-19

**Authors:** Xinhui Zhou, Hao Wang, Cuina Wang, Chao Zhao, Qian Peng, Tiehua Zhang, Changhui Zhao

**Affiliations:** ^1^ Department of Food Science and Engineering College of Food Science and Engineering Jilin University Changchun China; ^2^ Department of Food Quality and Safety College of Food Science and Engineering Jilin University Changchun China

**Keywords:** digestion, edible oil, nanoemulsion, whey protein

## Abstract

Beta‐carotene, the main dietary source of provitamin A, is required for maintaining optimum human health. The bioaccessibility of beta‐carotene can be greatly improved when ingested with fat. Therefore, the aim of the current study was to select proper oils (palm oil, coconut oil, fish oil, and corn oil) as a carrier to form stable nanoemulsion that can effectively enhance the bioaccessibility of beta‐carotene. The nanoemulsion was formulated with 90% (v/v) aqueous solution (2% whey protein isolate, WPI, w/v) and 10% (v/v) dispersed oil. The in vitro digestion experiment of nanoemulsions showed that the bioaccessibility of beta‐carotene was as followed in order: palm oil = corn oil > fish oil > coconut oil *(p *<* *0.05). The particle size of the nanoemulsion (initial particle size = 168–185 nm) was below 200 nm during 42 days’ storage at 25°C. The retention rates of beta‐carotene in nanoemulsions were 69.36%, 63.81%, 49.58%, and 54.91% with palm oil, coconut oil, fish oil, and corn oil, respectively. However, the particle size of the nanoemulsion increased significantly in the accelerated experiment at 55°C (*p *<* *0.05), in which the retention rates of beta‐carotene were 48.56%, 43.41%, 29.35%, and 33.60% with palm oil, coconut oil, fish oil, and corn oil, respectively. From above, we conclude that WPI‐stabilized beta‐carotene nanoemulsion with palm oil as the carrier is the most suitable system to increase bioaccessibility and stability of lipid‐soluble bioactive compounds such as beta‐carotene.

## 1 INTRODUCTION

Beta‐carotene, a kind of lipid‐soluble edible natural pigment, is widely used in food additives and feed supplements to prevent against vitamin A deficiency (Chen, Li, Li, McClements, & Xiao, 2017; Gutiérrez et al., 2013; Jain, Thakur, Ghoshal, Katare, & Shivhare, 2015). With high content of conjugated double bonds, carotenoids are very sensitive to environmental stresses during food processing and storage, such as heat, light, and oxygen. Additionally, the food matrix micro‐environment can also affect the stability of beta‐carotene (Xu, Wang, et al., 2013; Xu, Yuan, Gao, McClements, & Decker, 2013; Yi, Fan, Yokoyama, Zhang, & Zhao, 2016). To overcome these limitations, the nanoemulsion system has been developed for effectively stabilizing and delivering lipophilic nutraceuticals (Acosta, 2009). The mostly common oil‐in‐water emulsion can be obtained by solubilizing the lipophilic nutraceuticals within a carrier oil in the presence of certain surfactants followed by homogenization (McClements et al., 2016).

Bioaccessibility is a term to describe the amount of nutrients actually released from the food matrix ready for absorption from the gastrointestinal tract, while bioavailability has a broader sense that is to describe the portion of nutrients actually absorbed by the intestinal cell epithelium (McClements, Li, & Xiao, 2015). Apparently, good bioavailability of beta‐carotene requires high bioaccessibility that is greatly impacted by food matrix (Van LooBouwman et al., 2014). The bioaccessibility of beta‐carotene in emulsions depends on several factors including the particle size, oil composition, and emulsifier type (Rayner, 2015). Yi, Li, Zhong, and Yokoyama (2014) fabricated a range of emulsions of beta‐carotene with different particle sizes and found that the lipolysis and bioaccessibility increased with decreasing particle diameter. Salvia‐Trujillo, Qian, Martin‐Belloso, and McClements (2013b) investigated the influence of oil composition on the beta‐carotene bioaccessibility in emulsions by adjusting the proportion of long‐chain triglycerides (LCT) and medium‐chain triglycerides (MCT). As a result, high proportion of LCT enhanced the bioaccessibility of beta‐carotene. The bioaccessibility of beta‐carotene nanoemulsion prepared by digestible oil was higher than that in indigestible oil (Rao, Decker, Xiao, & McClements, 2013). Therefore, edible oils with high proportion of LCT are supposed to be a good oil carrier for improving beta‐carotene bioaccessibility. The stability of beta‐carotene emulsions can decrease with decreasing particle size at high temperature during storage (Yi, Zhang, Liang, Zhong, & Ma, 2015). Lipids, typically those with high degree of unsaturation, are susceptible to auto‐oxidation when dispersed in nanoemulsions due to increased surface area with reduced particle size (Yi et al., 2015). As a result, the lipophilic nutraceuticals carried in an oil‐in‐water system can deteriorate due to auto‐oxidation of the unsaturated fatty acids in the oil (Ghorbani Gorji, Smyth, Sharma, & Fitzgerald, 2016). Consistently, Liu, Gao, McClements, and Decker (2016) confirmed the positive correlation between lipid oxidation and the degradation of beta‐carotene in an emulsion. The fatty acids of oils from different food sources vary in their carbon chain length and the number of double bonds (Zhang, Zhang, Zhang, Decker, & McClements, 2015b). We hypothesized that beta‐carotene in nanoemulsions with highly saturated LCT oil should have both high chemical stability and bioaccessibility.

In the current study, whey protein was applied as the wall material to encapsulate beta‐carotene as a model molecule. WPI is a fraction of milk proteins that mainly consist of beta‐lactoglobulin and alpha‐lactalbumin. Compared with many large molecule emulsifiers, WPI has its unique advantages. For example, whey protein (a) has proper conformational, electrostatic, and amphiphilic nature (Fan, Yi, Zhang, Wen, & Zhao, 2017); (b) has good ability in binding metal ions and scavenging free radicals via amino acids such as tyrosine and cysteine (Yi et al., 2016); and (c) can effective improve the bioaccessibility of beta‐carotene in emulsions (Hou, Liu, Lei, & Gao, 2014).

We selected corn oil as a representative of the vegetable oil, fish oil as a representative of marine oil, and coconut oil and palm oil as representatives of saturated oils. The objective of this study was to investigate the effect of oil type on the physicochemical stability and the beta‐carotene bioaccessibility in WPI‐based emulsions.

## 2 MATERIALS AND METHODS

Palm oil (1.2% C_14:0_, 40.3% C_16:0_, 0.4% C_16:1_, 4.5% C_18:0_, 39.5% C_18:1_, 11.4% C_18:2_, 0.2% C_20:1_, 0.3% C_20:5_, and 0.7% C_22:6_), coconut oil (50.11% C_12:0_, 14.22% C_14:0_, 15.57% C_16:0_, 5.23% C_18:0_, 11.04% C_18:1_, and 3.02% C_18:2_), fish oil (3.2% C_14:0_, 7.8% C_16:0_, 2.6% C_18:0_, 0.6% C_20:0_, 0.4% C_22:0_, 3.9% C_16:1_, 0.7% C_16:2_, 6.1% C_18:1_, 0.8% C_18:2_, 2.0% C_20:1_, 0.3% C_20:2_, 2.5% C_22:1_, 27% C_20:5_, and 24% C_22:6_), and corn oil (11.2% C_16:0_, 2.2% C_18:0_, 28.9% C_18:1_, 55.5% C_18:2_, 1.1% C_18:3_, and 0.4% C_20:1_) were purchased from Huacheng Biological Corporation in Changchun, China. Whey protein isolate (WPI, 93.14%, w/w protein) was provided by Fonterra Co‐operative Group (Auckland, New Zealand). Beta‐carotene (97%, #22040), beta‐carotene standards (PHR1239), pepsin (porcine, P6887), bile extract (porcine, P8361), and pancreatin (porcine pancreas, P7545) were from Sigma‐Aldrich (St. Louis, MO). The methanol and acetonitrile of chromatographic grade were from Fisher Scientific (Fair Lawn, NJ). All other chemical reagents were purchased from Beijing Chemical Works (Beijing, China). The water was filtered through a Milli‐Q system (Millipore Corp. Bedford, MA).

### 2.1 Preparation of the beta‐carotene oil‐in‐water nanoemulsion

The WPI was dispersed in 10 mmol/l PBS buffer (pH = 7) containing 0.02% (w/v) sodium azide to a final concentration of 2% (w/v). To ensure full dissolution and hydration, the samples were placed at 4°C overnight. The oil phase was 0.3% (w/v) of beta‐carotene dissolved in oil. The oil solution and aqueous solution were then mixed (1:9 ratio) by centrifuge at 15,000 *g* for 2 min using a high‐speed blender (Avanti J‐E, Beckman Coulter, USA). The crude emulsion was then homogenized with a microfluidizer (M‐110L, Microfluidics, USA) for seven cycles at 15 kpsi.

### 2.2 Particle characterization

The particle size and its distribution of the emulsions were measured by dynamic light scattering using a Zetasizer Nano ZS (Malvern Instruments, Worcestershire, UK) as reported (Yi et al., 2016). The zeta potential of the particles was measured by phase analysis light scattering Zetasizer Nano ZS (Malvern Instruments, Worcestershire, UK) as reported (Salvia‐Trujillo et al., 2013b).

### 2.3 In vitro digestion

The in vitro digestion of the nanoemulsion samples was evaluated in a simulated gastrointestinal tract model with slight modifications (Yi et al., 2014). Briefly, 7.5 ml of the initial sample was diluted in twofold and 10 ml gastric juice (3.2 mg/ml pepsin and 0.15 M NaCl, pH: 2.0) was added. The mixture was incubated for 1 hr with continuous agitation at 100 rpm. After gastric digestion, the digesta was immediately adjusted to pH 7.0 using NaOH. Simulated intestinal juice (15 ml, containing 1.0 mg/ml pancreatin, 20.0 mg/ml bile extract, and 10 mmol/l CaCl_2_) was added and incubated for 2 hr with the pH maintained at 7.0 using an automatic titrator releasing 0.25 M NaOH drop by drop. Each experiment was repeated three times, with each in triplicate.

### 2.4 Analysis of beta‐carotene content

Beta‐carotene was extracted from emulsions using n‐hexane and ethanol, and quantitated by HPLC as described previously (Yi et al., 2014). In brief, 2.5 ml ethanol/n‐hexane solution (2:3) was added to 0.2 ml of the nanoemulsion in a centrifuge tube and vortexed for 30 s. The upper yellow supernatant was transferred to a brown glass flask. This process was repeated with 1.5 ml n‐hexane three times until the upper supernatant was clear. Then, the extract was diluted to 10 ml with n‐hexane in a brown glass flask and then subjected to HPLC 2010 liquid chromatography (Shimadzu, Tokyo, Japan). A C30 reverse‐phase analytical column (YMC Carotenoid, 250 4.6 mm i.d., 5 mm, YMC, Inc., Wilmington, NC) was applied. Operation conditions were as follows: Injection volume was 20 μl, the detection wavelength was 450 nm, and the flow rate was 1.0 ml/min. The composition of the mobile phase included solvents A and B. Solvent A was methanol: acetonitrile: H_2_O (84:14:2, v/v/v), and solvent B was dichloromethane. The solvent gradient elution program was 80A/20B to 45A/55B from 0 to 10 min, 45A/55B from 10 to 15 min, and 80A/20B from 15 to 20 min.

### 2.5 Bioaccessibility determination

The release property of beta‐carotene in the intestinal was measured as reported with slight modifications (Qian, Decker, Xiao, & McClements, 2012b). Briefly, 10 ml raw digesta was centrifuged at 2,500 *g* for 40 min at 25°C. After centrifugation, the digesta was separated into three layers: an opaque sediment phase at the bottom, a clear micelle phase in the middle, and an oily or creamed phase at top. Then, the aqueous micellar fraction was collected and filtered through a 0.22‐μm syringe filter to remove any insoluble beta‐carotene crystals. The filtrate was dried under a nitrogen stream and resuspended in 0.5 ml n‐hexane to be quantitated by HPLC. Each experiment was performed in triplicate.

### 2.6 Storage stability of beta‐carotene nanoemulsion

The nanoemulsion samples were stored at 25°C and 55°C in dark until the yellow color disappeared (Liu et al., 2016; Yi et al., 2015). The particle size and beta‐carotene retention were measured once a week. Each experiment was repeated three times, and every measurement was conducted in triplicate.

### 2.7 Confocal laser scanning microscopy fluorescence microscopy

The microstructures of emulsions were determined using confocal laser scanning microscopy (Zhang et al., 2016). In brief, 2 ml samples were mixed with 0.1 ml Nile red (0.125 mg/ml ethanol) and kept in dark at 4°C overnight. An appropriate amount of dyed sample was placed on a fluorescent measurement dish. The microstructure images were photographed and analyzed using the image analysis software (NIS‐Elements, Nikon, Melville, NY).

### 2.8 Statistical analysis

The data were analyzed by SPSS version 21.0 (SPSS Inc., Chicago, IL, USA). Values with *p *<* *0.05 were considered as significant. All figures were generated by Origin 8.0 (OriginLab Corporation, Northampton, MA).

## 3 RESULTS AND DISCUSSION

### 3.1 Impact of oil type on the physical properties of β‐carotene nanoemulsions

The mean particle size, the particle size distribution, and the microstructure of the initial emulsions were measured. All the emulsions showed a narrow distribution (polydispersity index [PDI] < 0.20; Table 1). The particle size of the emulsions carried with palm oil, coconut oil, fish oil, and corn oil were 168, 173, 185, and 177 nm, respectively (*p *<* *0.05; Table 1). The result indicated that the particle size of the initial emulsion was below 200 nm that met the requirement for nanoemulsion standard (Acosta, 2009). Under the same processing condition, the significant difference of particle size among three nanoemulsions was likely caused by different carbon chain length of the carrier oils. The previous study has reported that 16‐ and 18‐carbon fatty acids can be tightly combined within the hydrophobic site (Loch et al., 2013). Palm oil and corn oil are rich in 16‐ and 18‐carbon fatty acids which account for 80%, while fish oil is rich in 22‐carbon fatty acids and 20‐carbon fatty acids for approximately 40%. The corn oil nanoemulsion in this study had particle size in 177 nm, which was similar with the nanoemulsions produced using sunflower and olive oil which had abundant 16‐ and 18‐carbon fatty acids (Zhang et al., 2015b). On the other hand, Bengu et al. (Ozturk, Argin, Ozilgen, & McClements, 2015) fabricated emulsions of approximately 290 nm in size using the orange oil that was abundant in short carbon fatty acids. Our results indicated that vegetable oil rich in 16‐ and 18‐carbon fatty acids was a good option to be used as a carrier in oil‐in‐water nanoemulsions.

**Table 1:** Impact of carrier oil type on the particle size, distribution, and zeta potential of beta‐carotene nanoemulsions during in vitro digestion

	Initial emulsion			Gastric digesta			Intestinal digesta		
	Particle size	PDI	Zeta potential	Particle size	PDI	Zeta potential	Particle size	PDI	Zeta potential
Palm oil	168.43 ± 7.68^aA^	0.175 ± 0.012^aA^	−34.3 ± 0.36^aA^	175.78 ± 2.83^aA^	0.182 ± 0.012^aA^	−8.5 ± 0.18b^aB^	1248.88 ± 37.46^aB^	0.324 ± 0.051^aB^	−53.2 ± 0.72^aC^
Coconut oil	172.70 ± 1.91^bA^	0.144 ± 0.021^aA^	−25.4 ± 0.25^bA^	184.48 ± 6.98^bB^	0.231 ± 0.012^bA^	−3.2 ± 0.16^bB^	1119.00 ± 30.90^bC^	0.458 ± 0.040^bB^	−45.4 ± 1.07^bC^
Fish oil	184.97 ± 0.86^cA^	0.171 ± 0.013^aA^	−18.2 ± 0.19^cA^	255.89 ± 12.30^cB^	0.228 ± 0.015^bA^	−6.3 ± 0.58^cB^	1520.22 ± 70.87^cC^	0.293 ± 0.020^cB^	−29.2 ± 0.53^cC^
Corn oil	176.80 ± 0.44^dA^	0.188 ± 0.017^aA^	−29.6 ± 0.15^dA^	210.68 ± 14.90^dB^	0.211 ± 0.013^bA^	−5.4 ± 0.25^dB^	1290.29 ± 39.22^aC^	0.357 ± 0.024^aB^	−51.7 ± 0.41^aC^

Note. Different lowercase letters mean significant difference between various carrier oils (*p *<* *0.05). Different capital letters mean significant difference between in vitro digestion processes.

There was a significant difference in the zeta potential of the particles in the initial nanoemulsions of different oil types (−34.3, −25.4, −18.2, and −29.6 mV for palm oil, coconut oil, fish oil, and corn oil, respectively, *p *<* *0.05; Table 1). The pH of the initial nanoemulsion was well above the isoelectric point of WPI (pH = 5.0; Xu, Wang et al., 2013; Xu, Yuan, et al., 2013), and as a result, the nanoemulsion formed by WPI under neutral conditions had a high negative charge (Chang & McClements, 2016). Palm oil emulsion had a higher zeta potential compared with the emulsions fabricated with the other carrier oils, which was likely due to enhanced electrostatic repulsion and Van der Waals’ force between palm oil droplets (Loch et al., 2013). The ionic impurities such as free fatty acids, phospholipids, and/or mineral ions in oil phase can also move toward the particle interface due to electrostatic force and then alter the zeta potential (Qian et al., 2012b).

### 3.2 Impact of carrier oil on nanoemulsion physical stability in a digestion model

**Figure 1:**
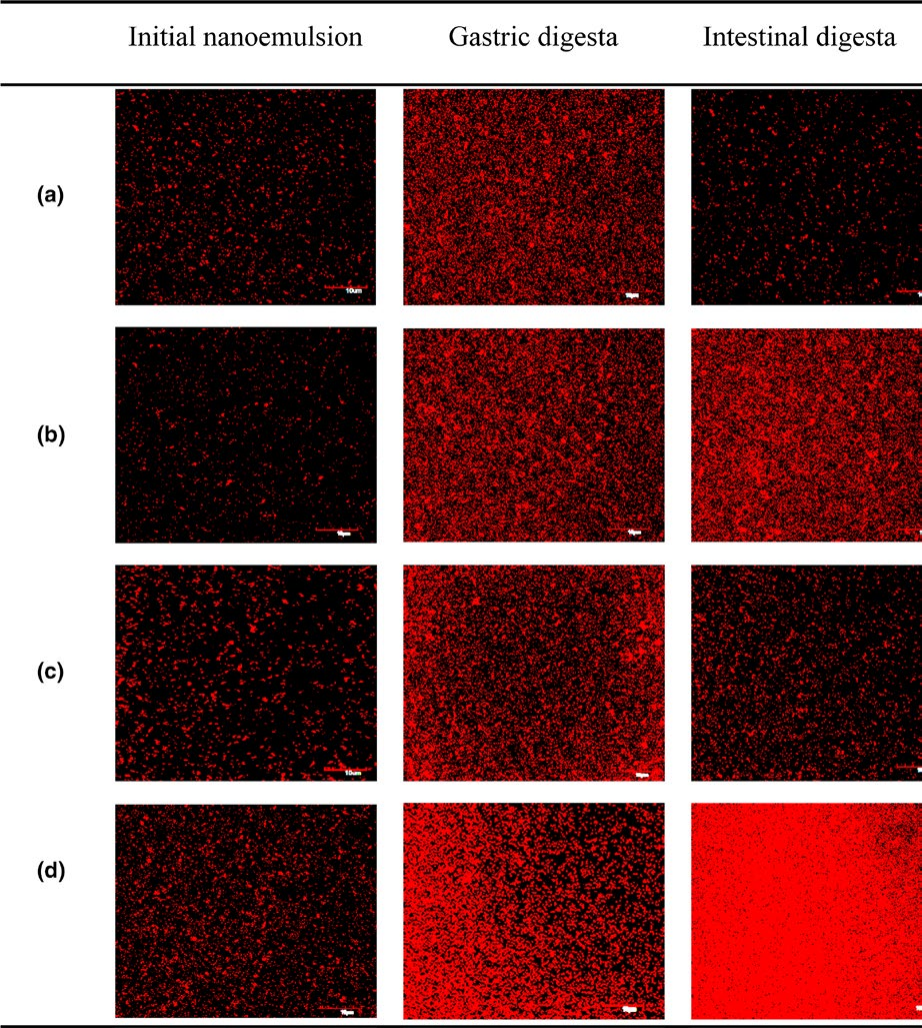
Microstructure of nanoemulsions with different oil compositions during in vitro digestion. (a) Nanoemulsion prepared by palm oil; (b) nanoemulsion prepared by coconut oil; (c) nanoemulsion prepared by fish oil; (d) nanoemulsion prepared by corn oil. Scale bar is 10 μm

After incubation in artificial gastric juices, the samples still had monodisperse particle distributions. With the exception of palm oil nanoemulsion, the particle size of the other nanoemulsions increased significantly (*p *<* *0.05; Table 1). There was evidence of some larger droplets formed after incubation of gastric juice in the emulsions with coconut oil, fish oil, and corn oil as seen in confocal microscopy images (Figure 1). This was consistent with the previous report of protein‐stabilized emulsions (Chang & McClements, 2016; Qiu, Zhao, Decker, & McClements, 2015). Lower pH and higher ionic strength weakened the nanoemulsion electrostatic repulsion evidenced by the decrease in the magnitude of the negative charges (Li, Li, Shen, Niu, & Fu, 2016; Ozturk et al., 2015; Qiu et al., 2015). Additionally, pepsin partially hydrolyzed some proteins (like lactalbumin and lactoferrin) surrounding the oil and thus altered the stability of the droplets to coalescence (Chang & McClements, 2016; Qiu et al., 2015). Interestingly, there was little change in the particle size and PDI of the palm oil nanoemulsion during incubation in gastric juice. This was likely because of its tight binding of the β‐lactoglobulin and palm oil which protected the nanoparticles from pepsin degradation. Compared with other large (sodium casein and denatured starch) or small molecular (Tween‐20, ML750) emulsifiers, WPI is more effective to protect beta‐carotene (Mao, Yang, Xu, Yuan, & Gao, 2010; Mun, Kim, & McClements, 2015; Zhang, Zhang, Zhang, Decker, & McClements, 2015a) because of the high content of beta‐lactoglobulin (~50%) that highly resists pepsin hydrolyzation (Hur, Decker, & McClements, 2009).

All of the digested emulsions showed a lower negative charge after exposure to the artificial gastric juices. Interestingly, the zeta potential of the emulsions (pH = 2) was close to zero. We did not observe a similar result as previous reports that the nanoemulsion at pH = 2 had a positive charge (Fan et al., 2017; Hur et al., 2009). Presumably, the interfacial composition and properties had changed due to partial hydrolysis of the WPI, leading to some peptides and free fatty acids released and absorbed to the oil droplets (Qiu et al., 2015).

After exposure to small intestine juices, most of the lipids were digested by pancreatin evidenced by disappearance of the dyed fat molecules in the digesta (Figure 1). The particle size increased drastically after intestinal incubation, which was partially attributed to aggregation of WPI. WPI was then be hydrolyzed by trypsin at pH = 7 (Table 1; *p *<* *0.05; Teo et al., 2016). Proteolyzed WPI thus led to breakdown of the oil‐in‐water structure of the nanoemulsion (Hou et al., 2014; Qiu et al., 2015; Xu, Yuan et al., 2013). The oil droplet was hydrolyzed after exposure to pancreatin. Then, beta‐carotene was then released and surrounded with anionic biosurfactants such as bile salts, phospholipids, and free fatty acids to form micelles for intestinal absorption (Acosta, 2009; Bibi, Holm, & Bauer‐Brandl, 2017; Liu, Hou, Lei, Chang, & Gao, 2012). The particle size distribution (60–1,600 nm) became wider. The large particles were the undigested lipid droplets, insoluble calcium, and precipitated beta‐carotene crystal in intestinal digesta (Ozturk et al., 2015), and the small particles were the bile salts vesicles possibly carried with beta‐carotene (Hernell, Staggers, & Carey, 1990; Netzel et al., 2011).

The digested emulsions from palm oil, coconut oil, and corn oil showed a relatively high negative charge (−45 to −58 mV) after exposure to small intestine juices (Table 1), possibly due to the existence of anionic species such as bile salts and phospholipids in small intestine juices (Qian et al., 2012b). The free fatty acids released from lipid digestion contributed to formation of anionic species that further increased the negative charge (Salvia‐Trujillo, Qian, Martin‐Belloso, & McClements, 2013a). However, the fish oil emulsion showed a relatively lower zeta potential after digestion, which was possibly because that the free fatty acids were not fully released as a result of its low degree of lipolysis under intestinal conditions (Komaiko, Sastrosubroto, & McClements, 2016; Walker, Decker, & McClements, 2015).

### 3.3 Impact of carrier oil on in vitro bioaccessibility

**Figure 2:**
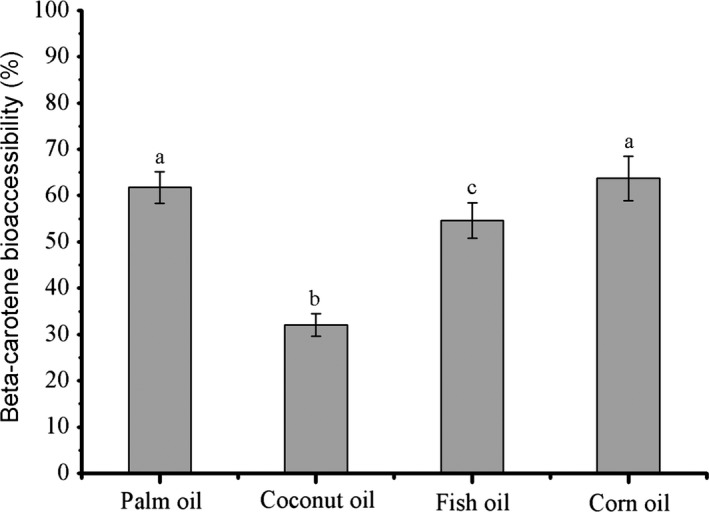
Impact of carrier oil type on the bioaccessibility of beta‐carotene in nanoemulsions. Different lowercase letters mean significant difference between various carrier oils (*p *<* *0.05)

The beta‐carotene bioaccessibility has been successfully measured in vitro digestion model (Hur et al., 2009; Karthik & Anandharamakrishnan, 2016; Liu et al., 2012; Majeed et al., 2016; Verrijssen et al., 2015). Our results showed that the bioaccessibility of the beta‐carotene decreased in the following order: palm oil (LCT) = corn oil (LCT) > fish oil (LCT) > coconut oil (MCT; Figure 2). This was probably because long‐chain fatty acids preferred to accumulate at the oil–water interface to form micelles, while medium‐chain fatty acids were water dispersible and easy to move into the surrounding aqueous phase (Qian et al., 2012b; Salvia‐Trujillo et al., 2013b). Additionally, LCT forms mixed micelles with relatively large hydrophobic domains suitable to deliver beta‐carotene (Zhang et al., 2016). However, the bioaccessibility of fish oil carried beta‐carotene was significantly lower than corn oil or palm oil, which was probably because of its special lipid composition (Komaiko et al., 2016; Ulven et al., 2011; Walker et al., 2015). Qian et al., (2012b) applied orange oil abundant of short‐length carbon chain to form beta‐carotene nanoemulsion, in which the bioaccessibility of beta‐carotene was close to zero. All the results supported our hypothesis that the length of carbon chain in the carrier oil plays an important role on the bioaccessibility of beta‐carotene emulsion. It is worthy to be noted that the beta‐carotene bioaccessibility increased with the particle size decreasing. The initial particle size of the emulsion tested in the current study was all below 200 nm. Under this situation, the lipid composition is supposed to be the main factor for bioaccessibility.

### 3.4 Impact of carrier oil type on the stability during storage

**Figure 3:**
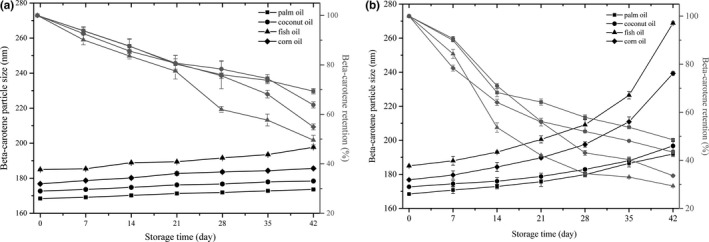
Impact of carrier oil type on the particle size and beta‐carotene retention of nanoemulsion at different temperatures. Changes of particle size and retention rates at 25°C (a) and 55°C (b) during storage

Nanoemulsion provided a large surface area that enhanced the bioaccessibility, but might also facilitate degradation of beta‐carotene during storage (Xu, Wang et al., 2013; Xu, Yuan et al., 2013). Lipid oxidation reactions are generally initiated at the interface between the oil and water, where the droplet surface hydrophobic nutraceutical molecules are easier to degrade when dispersed in nanoemulsions (Ghorbani Gorji et al., 2016). After 42 days of storage, no apparent flocculation or coalescence was observed in any sample tested. The particle size of the samples stored at 25°C slightly increased (*p *>* *0.05; Figure 3). The beta‐carotene retention decreased in the following order: palm oil (69.36%) > coconut oil (63.81%) > corn oil (54.91%) > fish oil (49.58%). However, the particle size of the samples stored at 55°C significantly increased (*p *<* *0.05; Figure 3). Also, the beta‐carotene retention decreased in the following order: palm oil (48.56%) > coconut oil (43.41%) > corn oil (33.60%) > fish oil (29.35%; Figure 3). These results indicated that the beta‐carotene in unsaturated fish oil and corn oil nanoemulsion was highly susceptible to degradation. Our result was consistent with a previous report that beta‐carotene in semisolid oils was more stable than that in emulsions carried by liquid oil (Cornacchia & Roos, 2011). Furthermore, in 55°C accelerated experiment, the particle size of fish oil and corn oil increased abruptly after 28 days of storage (Figure 3). Therefore, high degree of unsaturated fatty acids (rich in corn oil and fish oil) was easy to be oxidized and accelerated the oxidation rate of beta‐carotene encapsulated in such oils (Liu et al., 2016; Qian, Decker, Xiao, & McClements, 2012a).

## 4 CONCLUSION

This study has extensively investigated the impact of oil type on the stability and bioaccessibility of beta‐carotene in WPI‐based emulsions. Palm oil (high content of 16‐ to 18‐carbon chain fatty acids) was shown to be the best carrier oil tested for WPI‐stabilized beta‐carotene nanoemulsion. Our nanoemulsion delivery system based on palm oil and WPI has high potential to increase bioaccessibility and stability of lipid‐soluble bioactive compounds.

## CONFLICT OF INTEREST

None declared.

## ETHICAL STATEMENT

The authors declare no conflict of interest exists. Human and animal testing is unnecessary in this study
